# Depletion of FKBP51 in Female Mice Shapes HPA Axis Activity

**DOI:** 10.1371/journal.pone.0095796

**Published:** 2014-04-23

**Authors:** Lianne Hoeijmakers, Daniela Harbich, Bianca Schmid, Paul J. Lucassen, Klaus V. Wagner, Mathias V. Schmidt, Jakob Hartmann

**Affiliations:** 1 Max Planck Institute of Psychiatry, Munich, Bavaria, Germany; 2 Center for Neuroscience, Swammerdam Institute for Life Science, University of Amsterdam, Amsterdam, The Netherlands; Sapienza University of Rome, Italy

## Abstract

Psychiatric disorders such as depressive disorders and posttraumatic stress disorder are a major disease burden worldwide and have a higher incidence in women than in men. However, the underlying mechanism responsible for the sex-dependent differences is not fully understood. Besides environmental factors such as traumatic life events or chronic stress, genetic variants contribute to the development of such diseases. For instance, variations in the gene encoding the FK506 binding protein 51 (FKBP51) have been repeatedly associated with mood and anxiety. FKBP51 is a negative regulator of the glucocorticoid receptor and thereby of the hypothalamic–pituitary–adrenal axis that also interacts with other steroid hormone receptors such as the progesterone and androgen receptors. Thus, the predisposition of women to psychiatric disorders and the interaction of female hormones with FKBP51 and the glucocorticoid receptor implicate a possible difference in the regulation of the hypothalamic–pituitary–adrenal axis in female FKBP51 knockout (51KO) mice. Therefore, we investigated neuroendocrine, behavioural and physiological alterations relevant to mood disorders in female 51KO mice. Female 51KOs and wild type littermates were subjected to various behavioural tests, including the open field, elevated plus maze and forced swim test. The neuroendocrine profile was investigated under basal conditions and in response to an acute stressor. Furthermore, we analysed the mRNA expression levels of the glucocorticoid receptor and corticotrophin release hormone in different brain regions. Overall, female 51KO mice did not display any overt behavioural phenotype under basal conditions, but showed a reduced basal hypothalamic–pituitary–adrenal axis activity, a blunted response to, and an enhanced recovery from, acute stress. These characteristics strongly overlap with previous studies in male 51KO mice indicating that FKBP51 shapes the behavioural and neuroendocrine phenotype independent of the sex of the individual.

## Introduction

Anxiety and mood related disorders such as major depressive disorder (MDD), are a major burden for society [1] affecting more than 1 billion people worldwide [2]. Women are more susceptible to develop these disorders than men, which may relate to sex-dependent gene x environment interactions [3]–[5].

Besides the contribution of sex-specific factors, stress is a major environmental risk factor for depression, whose effects in mammals are mainly mediated by the hypothalamus–pituitary–adrenal (HPA) axis [6], [7]. The secretion of glucocorticoids, the main end product of the HPA axis, is regulated by the activation of the mineralocorticoid receptor and glucocorticoid receptor (GR) in different brain regions, including the hypothalamus, pituitary and hippocampus [8]. An impairment of this negative feedback loop results in an aberrant stress response and is associated with the development of MDD and anxiety disorders [5], [9]–[12].

The co-chaperone FK506 binding protein 51 (FKBP51; encoded by the gene *fkbp5*) acts as an inhibitor of GR activity, determines GR binding affinity to glucocorticoids and thus determines the negative feedback sensitivity of the HPA axis [10], [13], [14]. Moreover, translocation of the GR into the nucleus induces activation of an intracellular ultra-short feedback loop by directly enhancing *fkbp5* gene transcription, thus ultimately inhibiting GR activity [15]–[17]. FKBP51 also acts as an inhibitor of other steroid hormone receptors, such as the progesterone and androgen receptors [16], [18], thereby potentially linking the stress effects to gonadal hormone signalling.

The involvement of FKBP51 in stress related disorders was first indicated by studies illustrating an association between multiple genetic variants of *fkbp5* and the risk of MDD and PTSD development in humans [15], [19]–[25]. This relationship was further investigated in animal models to determine the mechanistic role of FKBP51 in HPA axis functioning. In both rats and mice, mRNA expression and protein levels of FKBP51 in GR-rich brain regions can be induced by administration of the glucocorticoid agonist dexamethasone, corticosterone (CORT) or by subjecting animals to stress paradigms namely restraint, food deprivation or chronic mild stress [26]–[30]. Depletion of FKBP51 in males did not reveal robust behavioural differences under basal conditions [31]. However, the lack of FKBP51 in stressed mice led to enhanced stress-coping behaviour and a less vulnerable neuroendocrine state [31], [32]. To date, only male rodents have been investigated in relation to FKBP51 while the potential influence of gonadal hormones like oestrogens has been largely ignored.

Beyond regulatory function of FKBP51 on GR hormone affinity, FKBP51 lowers ligand affinity of other gonadal steroid receptors such as the progesterone receptor and androgen receptor [16], [18], [33]. Thus, FKBP51 functions not only as a regulator of glucocorticoid-induced effects, but also as a negative feedback regulator for progesterone and androgens in the brain. This implicates a possible sex-specific regulation of the HPA axis and behaviour in respect to FKBP51 [34], which has not been investigated yet in previous studies on FKBP51 mediated HPA axis activity. Moreover, the exclusion of female hormones in previous studies of FKBP51 in mice is unfortunate as the susceptibly to develop psychiatric disorders is mediated in a sex-dependent gene x environment fashion [35].

The predisposition of women to psychiatric disorders and the interaction of female hormones such as progesterone or oestradiol with both FKBP51 and GR, implicate a possible differential regulation of the HPA axis in female 51KO mice. Therefore, the aim of this study was to characterize female 51KO and wild type (WT) littermates under basal conditions, with regard to possible behavioural, neuroendocrine and physiological alterations relevant to mood disorders. In this regard, we subjected female 51KO and WT littermates to a series of behavioural tests, examining exploratory, cognitive, anxiety-related and stress-coping behaviour. Furthermore, measurements of CORT and ACTH circulating levels, physiological parameters and gene expression levels were taken to assess the neuroendocrine profile and physiological differences in these animals.

## Materials and Methods

### Animals

Female wild type Fkbp51^+^/^+^ (WT) and knockout Fkbp51^-^/^-^ (51KO) mice of 3 to 4 months of age were used for the experiment. The 51KO mouse line was previously generated [36] and described [32]. Females were pair housed under standard housing conditions two weeks prior to the start of the experiment. Standard housing consisted of sufficient saw-dust bedding, cage enrichment, *ad libitum* access to food and water, a 12 h day-night cycle (lights on at 8:00 am) and constant temperature (23±2°C) conditions.

All animal experiments were performed in accordance to European Communities Council Directive 2010/63/EEC. All efforts were made to minimize animal suffering during the experiments. The protocols were approved by the committee for the Care and Use of Laboratory Animals of the Government of Upper Bavaria, Germany.

### Experimental design

Female 51KO mice (n = 14) and WT littermate controls (n = 14) were subjected to a battery of behavioural tests, conducted on 5 consecutive days in the following order: open field (OF), elevated plus maze (EPM), dark light box (DaLi), Y-maze and forced swim test (FST). Additionally, the FST served as acute stressor for a stress response test in order to determine CORT levels. The animals were sacrificed by decapitation 2 days after the FST. Trunk blood, brain, thymus and adrenal glands were collected for further analysis.

### Determination of estrous cycle phase

Determination of the estrous cycle phase of the females was performed by gentle flushing of the vaginal opening with 20 mL 7% standard saline solution, in order to collect vaginal cells. Samples containing the cells were transferred to a dry glass slide and coverslipped. The samples were viewed shortly after collection of the sample under the microscope at 400× magnification. Determination of the cycle phase was based on the presence and absence of leukocytes and appearance of epithelial cells, according to standard characteristics as described previously [37]. Ultimately, females were classified to be in estrous or non-estrous.

### Behavioural assessment

Behavioural tests were carried out between 08:00 am and 12:00 pm in the same room where the animals were housed. All behavioural tests have been described and validated previously [31], [32], [38]. They were performed using an automated video-tracking system (Anymaze 4.20, Stoelting, IL, USA). Collection of vaginal cells for determination of estrous cycle phase was performed daily after finalizing the behavioural test.

#### Open field (OF)

On day 1, general locomotor activity and exploratory behaviour was assessed in the OF for 15 min. Briefly, the apparatus consisted of an empty, enclosed, square arena (50×50×50 cm) made of grey polyvinylchloride (PVC), virtually divided into two areas, an inner zone (25×25 cm) and an outer zone. The arena was evenly and weakly illuminated (<10 lux). Parameters of interest included total distance travelled and inner zone time.

#### Elevated plus maze (EPM)

On the second day, anxiety-related behaviour was investigated using the EPM. The animal was placed in an elevated (50 cm) plus-shaped platform made of grey PVC, with two opposing open arms (30×5×0.5 cm) and two opposing enclosed arms (30×5×15 cm), which are connected by a central area (5×5 cm). Illumination was less than 10 lux in the enclosed arms and 25 lux in the open arms. Animals were placed in the center zone facing one of both closed arms at the beginning of a 10 minute trial. Parameters of interest included the distance travelled and relative open arm time.

#### Dark light box (DaLi)

The DaLi test is an additional setup to measure anxiety-related behaviour and was performed on day 3. Briefly, the apparatus consisted of a rectangular box with two compartments, the dark compartment (15 cm×20 cm×25 cm, lit with <10 lux) and the larger, more aversive and brightly lit compartment (30 cm×20 cm×25 cm, lit with approximately 700 lux), connected by a 4 cm long tunnel. Animals were placed in a corner of the dark compartment at the beginning of the test. Exploration of dark and lit compartment was allowed during a 10 minute period. The mice were considered to have entered the lit compartment after entering the zone with the hind paws. Behaviour of the animal was scored manually. Parameters of interest were latency to first lit compartment entry, number of entries and time spent in the lit compartment.

#### Y-maze

On day 4, hippocampal-dependent spatial memory was investigated in the Y-maze. The grey PVC apparatus consists of a platform of three identical arms, 30 cm long, 10 cm wide, and sides 15 cm high, connected by a center zone. The angle between each arm is 120°. The inner walls of each arm are marked with distinct, easy recognizable visual cues, using white adhesive tape. The illumination was 15 lux in all three arms. The test comprises two trials, the acquisition and retrieval trial. During the acquisition trail, one arm was blocked with a grey PVC board. The board is removed during the retrieval trail and this arm is further referred to as the novel arm. At the start of trial one, the mouse is placed into the central zone facing of one of both accessible arms and is allowed to explore the apparatus for a duration of 10 minutes. After 30 minutes inter-trial time spent in their home cage, the animals were reintroduced to the apparatus by placing them in the same starting position. In this retrieval phase, the mouse was allowed to explore all three arms for 5 minutes. Spatial memory performance was assessed by analysis of the distance travelled in the novel arm compared to distance travelled in the other arms as well as the number of entries into the novel arm.

#### Forced swim test (FST)

On the fifth day, analysis of escape-orientated behaviour and stress coping was investigated during the six minute FST trial. The animals were placed in a two litre glass beaker (diameter: 13 cm, height: 24 cm) filled with tap water (21°C) to a height of 15 cm, so that the mouse could not touch the bottom of the beaker with its hind paws or tail. After the test trial, the animals were dried with a towel and put back into their home cage. Scoring of animal behaviour was performed by an experienced observer who was blind to the genotype of the animals, and who classified the following behavioural patterns: floating, swimming and struggling.

### Sample processing

Females were sacrificed in the beginning of the light phase (between 08.00 and 10.00 am), 2 days after the last behavioural test (FST). After quick body weight measurement and anaesthesia by isoflurane, the animals were sacrificed by decapitation. Collection of vaginal cells was performed after sacrifice of the animal to minimize the time handling the animal prior to sacrifice. Brains were removed, snap-frozen in isobutene at −40°C and stored till further use at −80°C for *in situ* hybridization. Thymus and adrenal glands were removed, dissected from fat and weighted. Trunk blood was collected in 1.5 ml EDTA-coated tubes (Kabe Labortechnik, Germany) for basal CORT and ACTH level determination. All blood samples were kept on ice until centrifugation for 15 minutes at 8000 rpm and at 4°C. Blood plasma was transferred to new tubes; 100 µl blood plasma for ACTH analysis and 10 µl blood plasma for CORT analysis, stored at −20°C until further usage.

### Investigation of endocrine profile

The FST was used as an acute stressor in order to determine the stress response by measuring CORT blood plasma concentrations. After the FST, all mice were placed into their home cage to recover from the acute stressor. Blood samples for the stress response were collected by a tail cut 30 minutes (stress response) and 90 minutes (stress recovery) after the onset of the FST [39]. Blood was collected in 1.5 ml EDTA-coated tubes (Kabe Labortechnik, Germany) and processed as described above.

CORT and ACTH concentrations were determined by radioimmunoassay using a Corticosterone double antibody ^125^I RIA kit (sensitivity: 12.5 ng/ml, MP Biomedicals Inc) and Adrenocorticotropic double antibody hormone ^125^I RIA kit (sensitivity: 10 pg/ml, MP Biomedicals Inc) and were used following the manufacturers' instructions. Radioactivity of the pellet was measured with a gamma counter (Packard Cobra II Auto Gamma; Perkin-Elmer). Final CORT and ACTH levels were derived from the standard curve.

### In-situ hybridization

Brain tissue was sectioned at −20°C in a cryostate microtome at 18 µm in the coronal plane. Sections were thaw mounted on Super Frost Plus slides, air dried at a 28 °C warming plate and stored at −20°C. Hippocampal sections were further divided in dorsal (bregma >−2.70 mm) and ventral (bregma <−2.70 mm).

Expression levels of the GR and corticotrophin release hormone (CRH) genes were investigated by *in-situ* hybridization of different brain regions, dorsal hippocampus, ventral hippocampus and paraventricular nucleus (PVN) of the hypothalamus, with ^35^S UTP labelled ribonucleotide probes as previously described [40]. Briefly, sections were fixed in 4% para- formaldehyde and acetylated in 0.25% acetic anhydride in 0.1 M triethanolamine/HCl. Subsequently, brain sections were dehydrated in increasing concentrations of ethanol and treated with 100% chloroform. The antisense cRNA probes were transcribed from a linearized plasmid. Tissue sections were saturated with 100 ml of hybridization buffer containing approximately 3–5 million cpm ^35^S labeled riboprobe. Brain sections were coverslipped and incubated overnight at 55 °C. The following day, the sections were rinsed in 2×SSC solution containing 20 mg/l RNAse A and washed in increasingly stringent SSC solutions at room temperature. Finally, sections were washed in 0.1×SSC for 1 h at 65 °C and dehydrated through increasing concentrations of ethanol. The slides were exposed to Kodak Biomax MR films (Eastman Kodak Co., Rochester, NY) and developed. Autoradiographs were digitized, and expression was determined by optical densitometry utilizing the freely available NIH ImageJ software. The mean of four bilateral measurements (PVN) or two unilateral measurements (hippocampal subregions) was calculated for each animal, subtracting the background signal of a nearby structure not expressing the gene of interest from the measurements.

### Statistical analysis

Data graphs are presenting the means ± standard error of the mean. Analysis was performed using the commercially available software SPSS 17.0. Significance was accepted with P<0.05. All data were tested for outliers using the Grubbs' test (GraphPad software).

If the equality of variance was violated in Levene's test (P<0.05), the modified t-values were used for parametric measurements.

Body weight, thymus, adrenal glands and gene expression data were analyzed with the unpaired Student's t-test (independent factor genotype). The non-parametric Mann-Whitney test was applied in case of non-normality of these datasets.

The cycle phase (estrous or non-estrous) was assessed after each behavioural test to correct for the estrous cycle phase dependent effects on behavioural parameters and neuroendocrine levels. Behavioural and neuroendocrine data were analyzed using the analysis of covariance (ANCOVA) model with genotype as independent factor and the estrous cycle as covariate. Data of Y-maze novel arm entries were analysed after natural logarithmic transformation to meet the assumption of normality for statistical analysis.

## Results

### Physiological parameters of female 51KO mice

**Figure 1:**
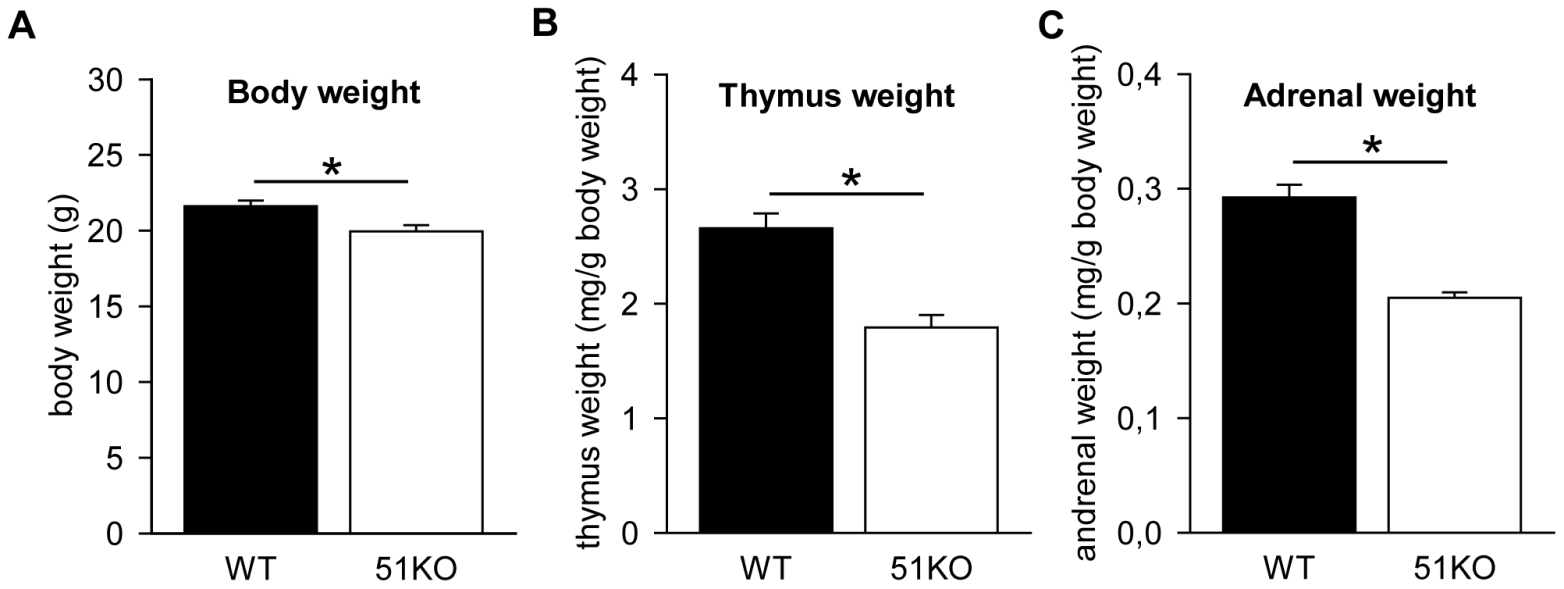
Physiological parameters of the female 51KO mice and WT littermates. (A) Bodyweight in 51KOs is reduced. (B) The thymus and (C) adrenal glands weights are reduced in 51KOs. * P<0.05.

Body weight measurements were taken before sacrifice and used for normalization of the organ weights. Student's t-test analysis detected that body weight of female 51KOs was significant lower than of their WT litter mates (*t_26_ = 3.026, P = 0.006*, fig 1A). In addition, both relative adrenal glands weight (Student's t-test: *t_25_ = 7.025, p<0.001*, fig 1B) and relative thymus weight (Mann-Whitney: *U = 10.00*, *p<0.001*, fig 1C) were reduced in the female 51KO mice in comparison to the WT females.

### Neuroendocrine profile

The endocrine profile of the females was investigated by analysis of the CORT blood plasma levels after an acute stressor and CORT and ACTH blood plasma levels under basal conditions.

**Figure 2:**
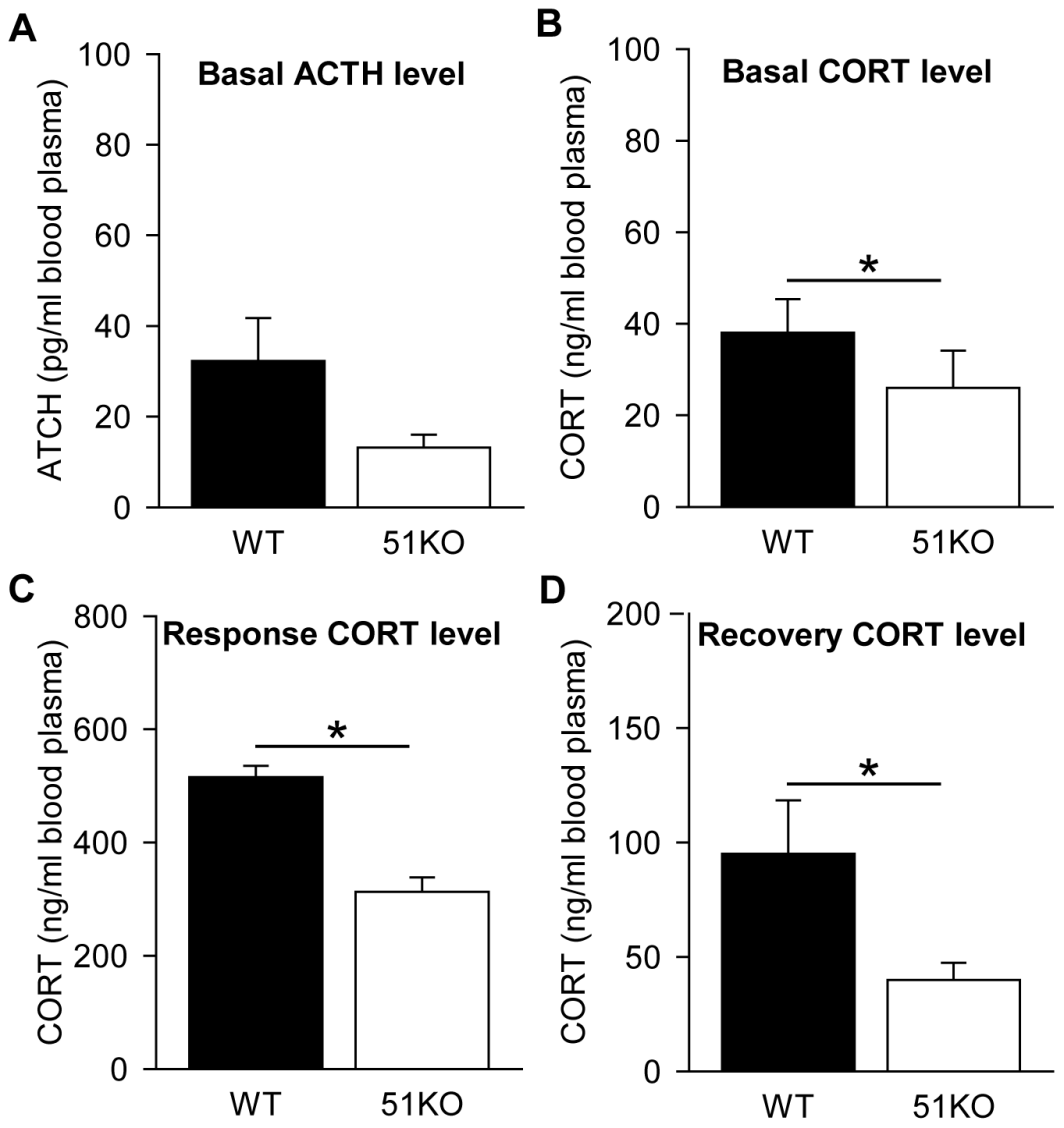
The neuroendocrine profile of female mice under basal conditions and after an acute stressor (FST). (A) ACTH levels under basal conditions are similar in the females. CORT levels in 51KOs are lower (B) under basal conditions, (C) 30 minutes after the onset of the FST (response) and (D) 90 minutes after the onset of the FST (recovery). * P<0.05.

Measurements under basal conditions were made using trunk blood at sacrifice. The ACTH levels in blood plasma did not differ between female WT and 51KO mice under basal conditions (ANCOVA: *F_1_ = 2.583, P = 0.121*, fig 2A). ANCOVA indicated lower CORT levels under basal conditions in female 51KOs (*F_1_ = 6.472, P = 0.018*, fig 2B). Upon stress, as induced by the FST, ANCOVA indicated an effect of genotype (*F_1_ = 35.514, P<0.001*). The CORT response levels of female WTs were elevated in comparison to the female 51KOs (fig 2C). In addition, ANCOVA revealed that CORT levels of female 51KOs were lower than of female WTs 90 minutes after the onset of the FST (*F_1_ = 5.392, P = 0.029*, fig 2D).

### Behavioural characterization of female 51KO mice

Female WT and 51KO mice were subjected to a series of 5 behavioural tests (OF, EPM, DaLi, Y-maze, FST) to investigate locomotion, anxiety and stress-coping behaviour as well as cognitive function.

**Figure 3:**
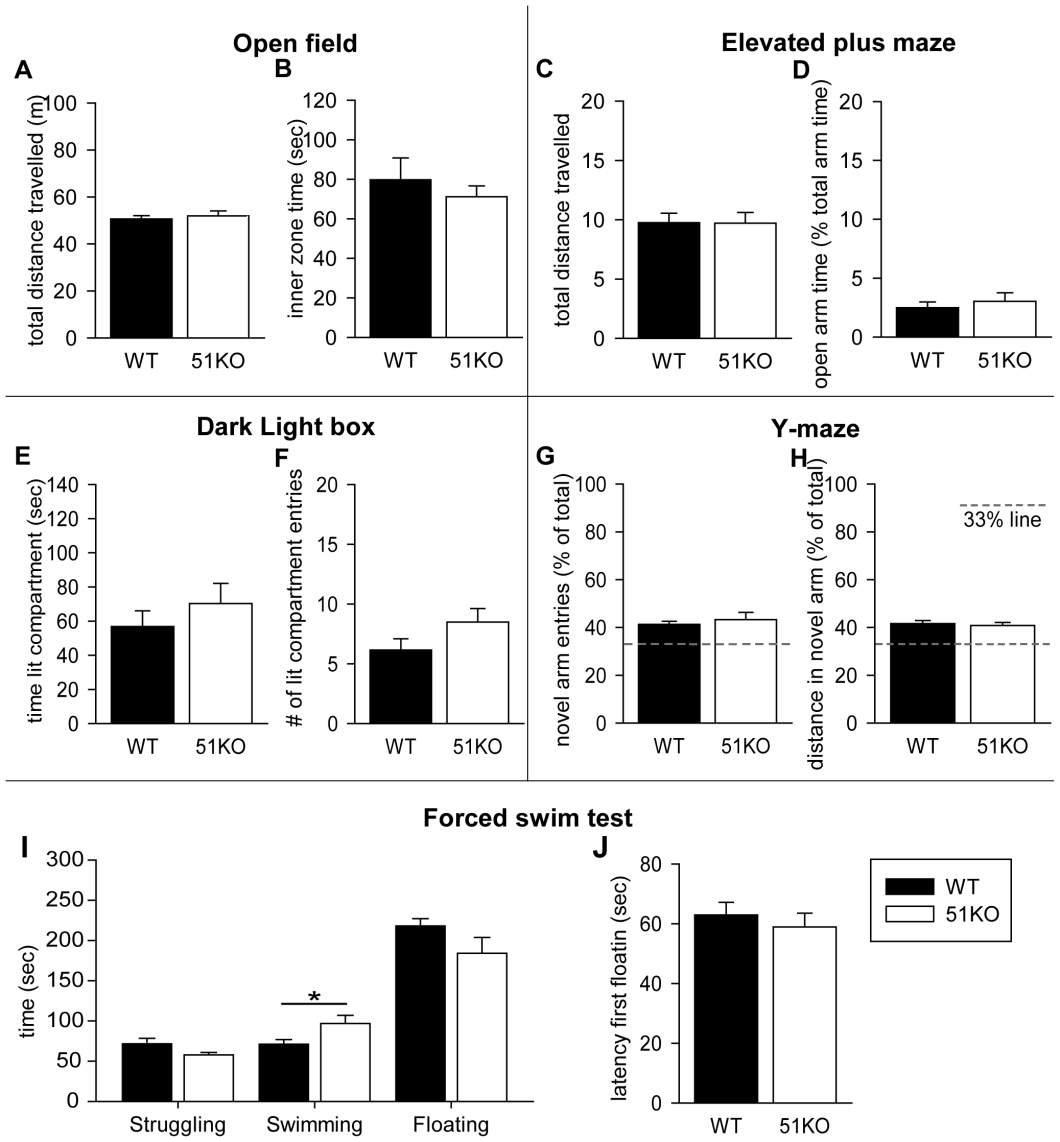
Behavioural profile of female 51KO and WT. Overall, female 51KOs and WTs performed similar in all presented behavioural tests. Open field: (A) Total distance travelled and (B) time in inner zone. Elevated plus maze: (C) Total distance travelled and (D) percentage of the relative time in the open arm. Dark light box: (E) Time in the lit compartment and (F) number of entries to the lit compartment. Y-maze: (G) Percentage of novel arm entries of total arm entries and (H) the percentage of distance travelled in the novel arm. The striped lines in the graphs of the Y-maze represent the 33% change level of the novel arm measurements. Forced swim test: (I) Time spent struggling, swimming or floating and (J) latency till the first observed floating episode. * P<0.05.

Female 51KO and WT mice behaved similar in respect to exploratory behaviour during the test. ANCOVA did not reveal an effect of genotype on locomotion (*F_1_ = 0.238, P = 0.629*, fig 3A) or on the time spent in the inner zone of the arena (*F_1_ = 0.440, P = 0.513*, fig 3B).

During the EPM, analysis of anxiety-related behaviour using ANCOVA revealed no effects of genotype. No differences were present in the distance travelled in the EPM (*F_1_ = 0.001, P = 0.979887*, fig 3C) or the relative time spent in the open arm (*F_1_ = 0.448, P = 0.510*, fig 3D).

Animals were tested in the DaLi as an additional measurement of anxiety-related behaviour. ANCOVA revealed no differences in the time spent in the lit compartment by 51KO mice (*F_1_ = 2.993, P = 0.097*, fig 3E) or in the number of entries to the lit compartment between 51KO and WT (*F_1_ = 1.207, P = 0.283*, fig 3F).

Cognitive functioning of the animals was tested in the hippocampal dependent spatial memory related Y-maze. Female WT and 51KO mice did not perform differently on any of the parameters. ANCOVA revealed no genotype effect on the number of entries to (*F_1_ = 0.048, P = 0.829*, fig 3G) or the distance travelled in the novel arm (*F_1_ = 0.024, P = 0.879*, fig 3H). Both the distance travelled in the novel arm and the number of entries of the novel arm exceeded the chance level of 33% in all animals (fig 3H,I).

The female mice were subjected to the FST to assess stress-coping behaviour. Analysis showed an increase in the swimming time in female 51KOs (*F_1_ = 7.466, P = 0.012*), but revealed no genotype effects on the time spent struggling (*F_1_ = 0.018, P = 0.893*) or floating (*F_1_ = 0.981, P = 0.331*, fig 3I). In addition, ANCOVA of the latency till the first floating moment revealed no effect of genotype (*F_1_ = 0.670, P = 0.411*, fig 3J).

### Gene expression

**Figure 4:**
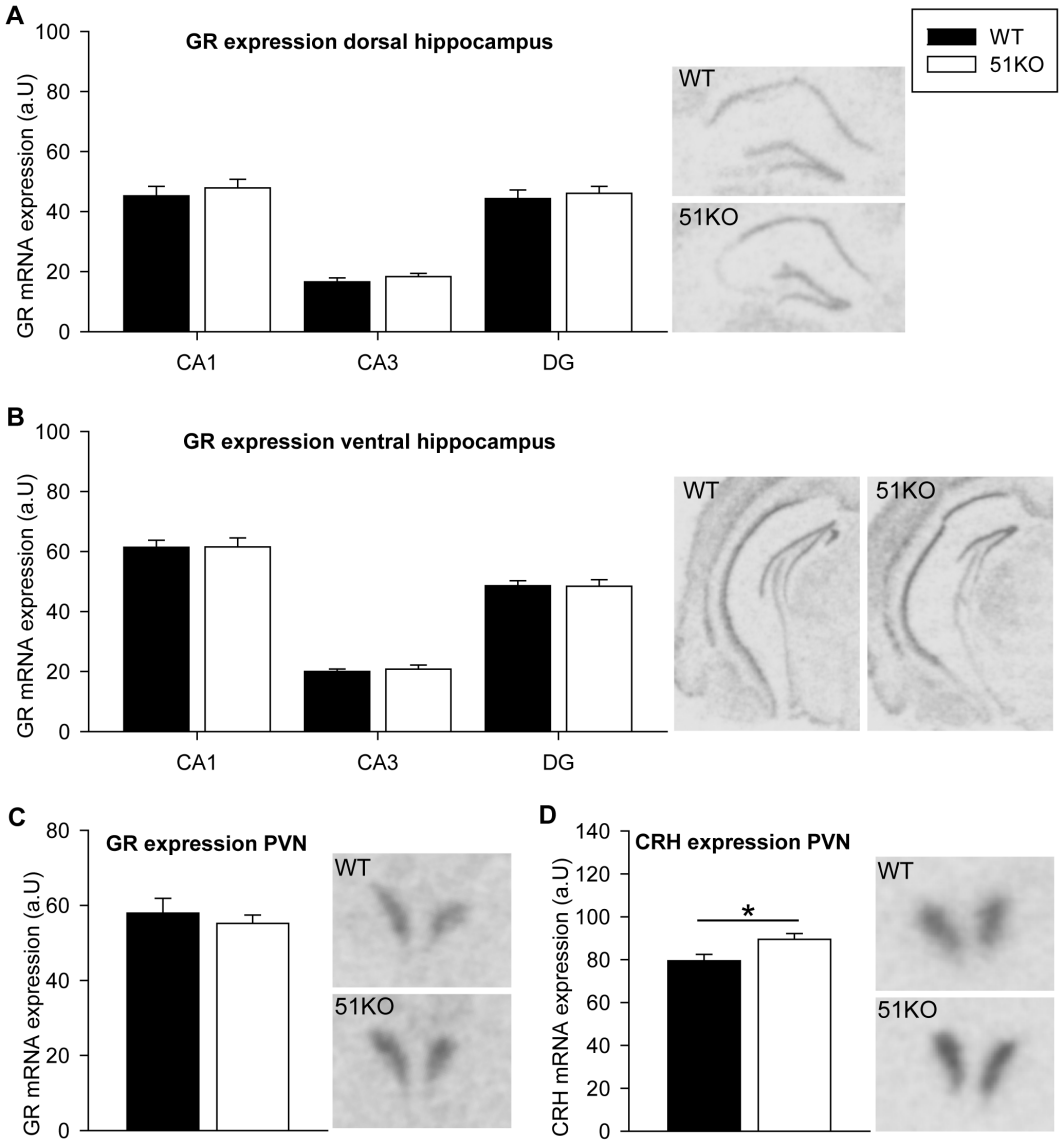
GR and CRH mRNA expression in the hippocampus and PVN. The hippocampus is split in different subregions of (A) the dorsal and (B) the ventral area for analysis of GR mRNA expression. (C) GR mRNA expression is similar in the PVN between genotype groups. (D) CRH expression in the PVN is elevated in the female 51KOs. Representative autoradiograph images are always depicted on the right side of the respective figure panel. * P<0.05.

Expression of GR mRNA in the hippocampus is similar in female 51KOs and WTs. Student's t-test analysis revealed no effect of genotype in the different subregions of the dorsal hippocampus (CA1: *t_26_ = −0.617, P = 0.542*, CA3: *t_26_ = −1.045, P = 0.306*, DG: *t_26_ = −0.466, P = 0.645*, fig 4A,B) or the ventral hippocampus (CA1: *t_26_ = −0.034, P = 0.973*, CA3: *t_21,257_ = −0.487, P = 0.631*, DG: *t_26_ = −0.064, P = 0.949*, fig 4C,D).

Student's t-test analysis of GR mRNA expression in the PVN showed no genotype effect (*t_21_ = 0.636, P = 0.532*, fig 4E,F), but revealed an increased level of CRH mRNA expression in the PVN of female 51KOs (*t_18_ = −2.529, P = 0.021*, fig 4G,H).

## Discussion

In this study, we investigated the effects of FKBP51 deletion in female mice on behavioural, physiological and neuroendocrine parameters. Overall, female 51KO mice presented a reduced basal HPA axis activity as well as a blunted response to, and an enhanced recovery from, an acute stressor. Nevertheless, 51KO female behaviour was not affected by the lack of FKBP51 under basal conditions. The physiological and endocrine profile of the female 51KOs differs from WT mice and is similar to that of male 51KO mice described previously.

### Physiological alterations in female 51KO mice

Similar to male 51KO mice, several physiological alterations, including reduced thymus and adrenal gland weights, are observed in female 51KOs compared to WT animals. Considering FKBP51 is known to reduce GR sensitivity in cellular models [41]–[43], an enhanced GR sensitivity most likely explains the thymus involution in female 51KO mice. Indeed, a study on mutant GR overexpressing rats shows an increased affinity for glucocorticoids in the thymus, resulting in strong thymocyte apoptosis, which can be reversed by adrenalectomy [44]. Along this line of reasoning, a report of adrenolectomized mice with a thymus-specific inducible GR transgene, showed a reduction in thymocyte number after induced GR overexpression, which could again be inhibited by a GR antagonist [45]. Chronic stress and HPA axis hyperactivity have been consistently associated with adrenal gland enlargement [32], [46], [47] with considerable consequences for brain structure and function [48]. However, we found that female 51KO mice showed rather decreased adrenal gland weight compared to WTs even under basal conditions. This may implicate attenuated HPA axis activity and enhanced negative feedback regulation in absence of FKBP51.

Next to the alterations in organ weights, body weight of the female 51KOs was also reduced, as it was in the males. Studies of obesity risk in psychiatric disorders relate enhanced glucocorticoid levels to the level of obesity [49], [50]. Hence, reduced circulating CORT levels and lower female 51KO body weight may point to the reversed situation, possibly as a result of the hypothesized GR sensitivity. The specific underlying biomechanism of the association between body weight and glucocorticoid levels is not identified to-date, and requires future research [31].

### FKBP51 depletion shapes levels of circulating CORT

The present profile of basal and stress-induced CORT secretion in female 51KO mice supports the hypothesis of an enhanced GR sensitivity. This could result from the depleted FKBP51 mediated inhibition of hormone binding to the GR. Under basal condition, as well as shortly after an acute stressor and during the recovery of this stressor, female 51KOs show reduced circulating CORT, thereby exhibiting a highly similar phenotype to male 51KO mice. However, ACTH levels do not show a significant modulation in female 51KOs at the present time point. It cannot be ruled out that over the course of the circadian rhythm (e.g. at the onset of the dark period) differences in ACTH secretion might be apparent. Overall, the neuroendocrine profile of the female 51KO mice is modulated in a similar manner as the previously described male 51KOs [31], [32]. Beyond its role on GR and thus HPA axis activation, it is known that FKBP51 inhibits gonadal hormone receptors in the brain [16], [18], [33], [34], suggesting that FKBP51 may have distinct effects in male and female mice. However, the presented neuroendocrine profiles of female and male 51KOs strongly overlap and no genotype x estrous cycle interaction was observed.

### Expression levels of GR and CRH

Next to the circulating glucocorticoids, expression of GR is a key factor affecting HPA axis feedback and regulation. GR mRNA levels in the PVN of the hypothalamus and in different subregions of the hippocampus were similar in female 51KOs and WT animals. Interestingly, the observed increase in CRH mRNA in the female 51KOs, might be due to a reduced GR-mediated negative feedback on CRH expression as a consequence of the lower circulating CORT levels in these animals. In male 51KOs, no differences of CRH mRNA in the PVN are described [32], indicating a sex-specific difference in HPA axis regulation at the level of the PVN in 51KO animals. Overall, possible compensatory mechanisms in the 51KO females might involve other components of the HPA axis or steroid hormones. At least in human brain, sex specific differences in GR protein expression in depression are present [51].

### FKBP51 deletion in female mice does not affect behaviour under basal conditions

In order to determine the behavioural profile of female mice depleted of FKBP51, mice were subjected to a battery of behavioural tests. The behavioural phenotype of female 51KO mice appears to be unaffected by the depletion of the FKPB51 under basal conditions. Female 51KOs perform adequate and similar to WT littermates, in all performed behavioural tests. The increase in swimming time during the FST is the only difference between 51KOs and WTs. This type of behaviour is considered to be neutral, whereas no differences were observed in the determinative types of behaviour during this test (i.e. attempting to escape and despair-like types of behaviour). Overall the behaviour of female 51KOs is in line with previous studies in male 51KOs that also did not display behavioural alterations under basal conditions. Yet, FKPB51 deletion led to marked alterations in males that were challenged by chronic stress [31], [32]. Whether this is the case for female mice as well remains to be elucidated.

In accordance to the regulation of CORT and ACTH in female 51KOs, no behavioural differences under basal conditions or in stress-coping behaviour during the FST were observed nor could they be subscribed to the effects of gonadal hormones in female 51KOs and WTs. Although FKBP51 interacts with steroid hormones besides CORT [16], [18], [33], [34], no interaction of FKBP51 with the estrous cycle was present in the current study. Future investigation is needed to reveal whether this is only the case under basal conditions. Studies of chronic stress models in female mice at least illustrate regulatory effects of oestrogens and androgens on HPA axis activity and behaviour [52]–[54]. Therefore, even though no interaction of FKBP51 in female 51KOs is indicated in the present study, it is possible that mediating effects of female gonadal hormones in absence of FKBP51 are present under other conditions, namely after exposure to chronic stress.

### Keynotes and future directions

The current study shows that a lack of FKBP51 in both male and female mice shapes the HPA axis reactivity but does not affect behaviour under basal conditions. This supports the use of both genders for the investigation of 51KO mice under stressful conditions to determine FKBP51's mediating effects on HPA axis functioning and stress-related disorder vulnerability. One limitation of the present study is that FKBP51 might also affect other pathways not mediated by the GR. Also, the choice for the 51KO mice, a conventional knockout mouse line, brings forward the possibility of compensatory mechanisms [17], [55] and it cannot be excluded this has contributed to the present data. Further investigation is needed to confirm the hypothesis that FKBP51 mediates GR sensitivity in this mouse line. However, the present results on the behavioural and neuroendocrine parameters in both the male and female 51KO mice, strengthen the choice for experimental designs in which both genders are included when studying (chronic) stress effects in these mice. Intriguingly, our results also indicate that FKBP51 shapes the neuroendocrine profile in a sex-independent manner, a finding that may help in the interpretation of future clinical studies implicating FKBP51 in the emergence of mood disorders.
